# Updates on mesenchymal stem cell therapies for articular cartilage regeneration in large animal models

**DOI:** 10.3389/fcell.2022.982199

**Published:** 2022-09-06

**Authors:** Timothy P. Liu, Pin Ha, Crystal Y. Xiao, Sang Yub Kim, Andrew R. Jensen, Jeremiah Easley, Qingqiang Yao, Xinli Zhang

**Affiliations:** ^1^ Department of Orthopaedic Surgery, David Geffen School of Medicine, University of California, Los Angeles, Los Angeles, CA, United States; ^2^ Division of Oral and Systemic Health Sciences, School of Dentistry, University of California, Los Angeles, Los Angeles, CA, United States; ^3^ Samueli School of Bioengineering, University of California, Los Angeles, Los Angeles, CA, United States; ^4^ Preclinical Surgical Research Laboratory, Department of Clinical Sciences, College of Veterinary Medicine and Biomedical Sciences, Colorado State University, Fort Collins, CO, United States; ^5^ Department of Orthopaedic Surgery, Nanjing First Hospital, Nanjing Medical University, Nanjing, Jiangsu, China

**Keywords:** cartilage regeneration, mesenchymal stem cell, large animal, osteochondral defect, tissue engineering, intraarticular injection, cell implantation, osteoarthritis

## Abstract

There is an unmet need for novel and efficacious therapeutics for regenerating injured articular cartilage in progressive osteoarthritis (OA) and/or trauma. Mesenchymal stem cells (MSCs) are particularly promising for their chondrogenic differentiation, local healing environment modulation, and tissue- and organism-specific activity; however, despite early *in vivo* success, MSCs require further investigation in highly-translatable models prior to disseminated clinical usage. Large animal models, such as canine, porcine, ruminant, and equine models, are particularly valuable for studying allogenic and xenogenic human MSCs in a human-like osteochondral microenvironment, and thus play a critical role in identifying promising approaches for subsequent clinical investigation. In this mini-review, we focus on [1] considerations for MSC-harnessing studies in each large animal model, [2] source tissues and organisms of MSCs for large animal studies, and [3] tissue engineering strategies for optimizing MSC-based cartilage regeneration in large animal models, with a focus on research published within the last 5 years. We also highlight the dearth of standard assessments and protocols regarding several crucial aspects of MSC-harnessing cartilage regeneration in large animal models, and call for further research to maximize the translatability of future MSC findings.

## Introduction

Articular cartilage injury secondary to trauma and aging affects over 300 million people (Safiri et al., 2020) and poses significant health burdens at the individual and socioeconomic level (Hunter et al., 2014; Katz et al., 2021). Injury ranges from acute defects, which can accelerate osteoarthritis (OA) if untreated, to diffuse cartilage loss in end-stage disease (Chen et al., 2017). Moreover, cartilage possesses particularly poor intrinsic healing capacity due to it’s avascularity, complex matrix architecture, and limited chondrocyte replication (Grassel and Aszodi 2019).

There exists an unmet need for novel and efficacious treatments for articular cartilage injury. Osteochondral grafting (OCG) and autologous chondrocyte implantation (ACI) restore the articular surface in focal defects, but suffer from donor site morbidity, limited tissue availability, and poor efficacy for diffuse cartilage loss (Zhang et al., 2021). Likewise, microfracture insufficiently recruits host growth factors and stem cells, promoting fibrocartilage formation with poor biomechanics (Mustapich et al., 2020). Total joint arthroplasty is indicated for end-stage OA in large joints, which alleviates pain but introduces potential complications including implant infection, postoperative stiffness and pain, and need for revision surgery (Heo et al., 2020). Therefore, no existing treatments effectively reconstitute the osteochondral unit.

Among emerging therapies, mesenchymal stem cells (MSCs) demonstrate promising potential for *in vivo* cartilage regeneration (Zhang et al., 2019). However, clinical translation has been limited despite multiple ongoing clinical trials for intra-articular MSC injection (Hernigou et al., 2021; Lamo-Espinosa et al., 2021). Arguably, the disconnected and inconsistent results between preclinical small and large animal studies may contribute substantially to the limited translation in current practice. Therefore, in this mini-review we discuss [1] highly clinically-relevant large animal models of cartilage regeneration, [2] source tissues and organisms for MSCs in large animal studies, and [3] tissue engineering strategies for optimizing MSC-based cartilage regeneration in large animals, focusing on research published within the last 5 years. We extracted relevant literature from Pubmed using the terms “mesenchymal stem cell,” “articular cartilage regeneration,” and either “pig,” “sheep,” “goat,” “dog,” or “horse.”

## Large animal models for MSC-based articular cartilage regeneration

Unlike the small joint size, thin articular cartilage, and robust cartilaginous defect regeneration of small animals (i.e., rodents, rabbits) (Libbin and Rivera 1989; Moran et al., 2016), large animal models are particularly useful for studying and identifying proposed treatments for further clinical investigation. Focal defects model isolated injury and regeneration, involve the full-thickness chondral layer or entire osteochondral unit on load- or non-load-bearing surfaces, and unlike in clinical disease, possess well-defined margins and cover limited areas (Cook et al., 2014). Alternatively, diffuse cartilage degeneration akin to OA is induced *via* articular surface surgical manipulation/injury (Zhang B. Y. et al., 2018), anterior cruciate ligament (ACL) and/or medial meniscus resection followed by weight-bearing (Ude et al., 2014), and chemical treatment (Uilenreef et al., 2019). Spontaneous OA is also studied in non-human primates (McCoy 2015) and long-lived companion animals including canines and equines, which can develop OA in their natural life course (Sasaki et al., 2019) (Table 1). Selectively-bred genetic OA is typically limited to small animal models (Chen et al., 2017).

**TABLE 1:** Advantages, disadvantages, and logistics of large animal models for articular cartilage injury and regeneration with MSC treatment, with parameters of relevant studies published within the last 5 years.

Animal model	Porcine	Goat	Sheep	Equine	Canine
Articular cartilage thickness	1–2 mm	1.5–2 mm	0.4–1.7 mm	1.5–2 mm	0.6–1.3 mm
Defect diameter	6–8 mm	6–10 mm	7–10 mm	6–20 mm	2–10 mm (4 mm most common)
Advantages	comparable biomechanics, comparable joint size	comparable biomechanics, comparable joint size, relatively inexpensive/easy to maintain	comparable biomechanics, comparable joint size, relatively inexpensive/easy to maintain	spontaneous OA, comparable biomechanics, comparable joint/cartilage size	spontaneous OA, relatively inexpensive/easy to maintain, compliant with postoperative exercise and loading regimens
Disadvantages	relatively late skeletal maturity, poor compliance with postoperative exercise/loading regimens, expensive and difficult to maintain	relatively late skeletal maturity, poor compliance with postoperative exercise/loading regimens, higher peak knee pressure	relatively late skeletal maturity, poor compliance with postoperative exercise/loading regimens	relatively late skeletal maturity, expensive and difficult to maintain, postoperative overloading, greater biomechanical load, strict licensing requirements	ethical concerns, limited noninvasive analysis methods
OA induction methods	ACL transection, partial/total meniscectomy, monosodium iodoacetate, chondral and osteochondral defect	partial/total meniscectomy, chondral and osteochondral defect	ACL transection, partial/total meniscectomy, chondral and osteochondral defect	spontaneous, osteochondral fragment, surgical impaction, chondral and osteochondral defect	spontaneous, ACL transection, partial/total meniscectomy, chondral and osteochondral defect
MSC Types	bMSCs, aMSCs, sMSCs, human bMSCs, human umMSCs	bMSCs, human umMSCs, human ubMSCs	bMSCs, aMSCs	bMSCs, sMSCs	bMSCs, aMSCs, umMSCs
MSC delivery route	Seeded onto implanted scaffolds, direct implantation	Intra-articular injection, seeded onto implanted scaffolds, direct implantation	Intra-articular injection, seeded onto implanted scaffolds, direct implantation	Seeded onto implanted scaffolds, direct implantation	Intra-articular injection
Injected MSC dose	n/a	25 million	2.5–50 million	n/a	1–10 million
Implanted MSC dose	0.4–30 million	1–60 million	2.5–30 million/ml	1–50 million	n/a
Length of Study	12–26 weeks	16–40 weeks	6–27 weeks	26–52 weeks	5–28 weeks
Treatment Outcomes	Lv et al. (2018): Improved gross/histological score, GAG content	Zhang et al. (2018a): Improved MRI/histological appearance, increased collagen II, compared to microfracture	Feng et al. (2018)—Improved MRI/histological scores, decreased synovial fluid inflammatory factors, thicker cartilage, allogenic MSC survival at least 14 weeks	Murata et al. (2022): improved radiographic defect filling, MRI/gross/histological scores	Li et al. (2018): improved radiographic defect filling, gross/histological scores
	Yamasaki et al. (2019): Improved MRI/histological score, increased radiographic defect filling	Zhang et al. (2020): Improved gross/MRI/histological appearance, higher GAG content and Young’s modulus, persistent xenogenic umMSCs in chondrocyte/MSC co-culture scaffold	Veronesi et al. (2022)—improved macroscopic/histological/synovial histological score, decreased local inflammatory markers, with stromal vascular fraction outperforming expanded MSCs		Zhang et al. (2018b): improved MRI X-ray appearance, thicker neocartilage, decreased circulating inflammatory markers
	Kondo et al. (2019): Improved gross/histological score, MRI appearance, only at study endpoint				
	Tseng et al. (2018): Increased defect filling, histological appearance, decreased fibrous neotissue	Kim et al. (2022): Improved gross/X-ray score, lameness score	Keller et al. (2019)—No inflammatory cell infiltrate, comparable histological scores for matrix staining, superficial/mid/deep zone, and overall assessment to autograft, at end-point	Chu et al. (2018): Similar gross/MRI/histological score and fibrocartilage formation for nonexpanded bone marrow concentrate and microfracture	De Francesco et al. (2021): improved lameness and pain scores, trend towards reduced synovial inflammatory markers
	Wu et al. (2019): Improved gross appearance, histological score. HA increased proliferation and cartilage-specific gene expression	Wei et al. (2019): Improved gross/histological scores	Vahedi et al. (2019)—Increased gross defect filling with cartilaginous tissue, increased expression of collagen II, aggrecan, and SOX9, for MSCs with scaffold	Mancini et al. (2020): limited cartilaginous tissue formation and persistent hydrogel on histology, for both bilayer constructs	
	Theruvath et al. (2021): Improved gross/MRI/histological score, collagen II content		Favreau et al. (2020)—Improved gross scores, MRI/histological appearance, regenerated cartilage surface area		
	Bothe et al. (2019): Erosion of bone, decreased histological score with biphasic scaffold implantation		Di Bella et al. (2019)—improved gross/histological scores, for MSCs in *in situ-*printed scaffolds but not MSCs in pre-printed scaffolds		

### Canine models

Domestic canines are unique, relatively long-lived models that suffer from similar spontaneous OA and poor cartilage healing as humans. Canines also handle postoperative exercise and loading regimens particularly well (Chu et al., 2010). Different breeds exhibit varying biomechanics, load patterns, and skeletal maturity ages based on size, which may complicate translation. Uniquely, the canine’s role as a family pet also presents ethical issues and limits the extent of post-treatment analysis, although arthroscopies can still rapidly enable articular surface evaluation (Chu et al., 2010).

Anatomically, canine cartilage is thinner than in humans and only relatively small defects, most commonly 4 mm, are created which limits comparability (Ahern et al., 2009). Like most quadrupeds, the canine knee joint also exhibits greater flexion and decreased extension than in humans, and possesses a quadrupedal-specific long digital extensor tendon which supplements joint stability (Proffen et al., 2012).

Allogenic and xenogenic human MSCs yield promising cartilage regeneration results in canines. Intra-articular injection of human umbilical cord matrix-derived MSCs (umMSCs) increased regenerated cartilage thickness and improved articular surface appearance on magnetic resonance imaging (MRI) (Zhang B. Y. et al., 2018). Adipose tissue-derived MSCs (aMSCs) and umMSCs injections also trended towards suppressed blood/synovial inflammatory markers, including interleukin-6 and tumor necrosis factor-alpha (Botto et al., 2022). Moreover, intra-articular knee injection of 10 million allogenic bone marrow-derived MSCs (bMSCs) and hyaluronic acid (HA) encouraged cartilaginous tissue formation in chondral defects on gross and histologic analysis, compared to HA or saline alone (Li et al., 2018).

### Porcine models

Miniature porcine breeds are among the most commonly-studied large animals, due to their similar joint size, loading mechanics, weight, intrinsically-poor cartilage regenerative ability, collagen fiber arrangement, bone apposition rate, and trabecular thickness as humans (Proffen et al., 2012; Takroni et al., 2016). Minipigs are also commonly used to evaluate inflammation and toxicity of implanted osteochondral biomaterials (Cone et al., 2017). At 1–2 mm thick, minipig cartilage is thinner than in humans; nevertheless, larger 6–8 mm diameter osteochondral defects can be created (Chu et al., 2010). Disadvantageously, minipigs cannot participate in many exercise or weight-bearing regimens. Like other large animals, only skeletally-mature minipigs exhibit diminished intrinsic cartilaginous repair, which extends maintenance duration and overall cost.

Numerous studies support that autologous, allogenic, and xenogenic human MSC administration bolsters osteochondral reconstitution in pigs. Porcine aMSCs seeded onto decellularized cartilage extracellular matrix (DCECM) performed similarly to chondrocytes regarding histological chondral defect regeneration (Lu L. et al., 2021). Scaffold-free implantation of porcine bMSCs and aMSCs at 5–30 million cells per defect, respectively, also improved cartilage histological and MRI score compared to nontreatment (Yamasaki et al., 2019; Theruvath et al., 2021). Likewise, autologous porcine synovial tissue-derived MSCs (sMSCs) aggregates, at 400,000 cells/defect, bolstered histology and macroscopic scores in femoral condyle after 12 weeks (Kondo et al., 2019).

Regarding xenogenic studies, human bMSCs seeded and chondrogenically-induced on collagen scaffold encouraged cartilaginous tissue formation 5 months after bone plug co-implantation into osteoarthritic pigs (Tseng et al., 2018). Moreover, 5 million human umMSCs suspended in HA hydrogel enhanced gross and histological cartilage scoring compared with nontreatment, in porcine full-thickness trochlear defects (Wu et al., 2019). Such studies suggest comparable therapeutic efficacy of human-derived and porcine MSCs in minipigs.

### Ruminant models

Ruminants are popular and accessible models for osteochondral defect studies, as they exhibit poor spontaneous regeneration and are cheaper and easier to handle than other large animals. Cyclic loading, biomechanics, and contact pressure during ruminant gait is comparable to that of humans (Moran et al., 2016). Biomechanically, goat knees experience higher peak pressures than in human tissues, which contributes to comparatively poor cartilage repair (Patil et al., 2014). Caprine defects up to 6 mm are reported, although cartilage thickness varies significantly across breed size/sex with upper limits of 1.5–2.0 mm. Ovine models possess a cartilage thickness of 0.4–1.7 mm, with defects up to 7 mm reported (Cook et al., 2014).

Numerous studies report that allogenic/xenogeneic MSCs promote osteochondral regeneration in ruminants. Allogenic bMSCs or aMSCs seeded onto polycaprolactone, collagen, alginate, and/or tantalum scaffolds improve cartilaginous tissue deposition and histologic scores compared to untreated defects in sheep (Vahedi et al., 2019; Favreau et al., 2020) and goats (Wei et al., 2019), with comparable histological scoring to autograft reported (Keller et al., 2019). Injection of aMSCs in sheep similarly suppressed synovial fluid inflammatory factors and bolstered histological, macroscopic, and MRI scores (Feng et al., 2018; Lv et al., 2018; Veronesi et al., 2022).

Regarding human-derived MSC studies, 1 million umMSCs seeded on DCECM scaffold yielded improved tissue elasticity modulus, collagen II content, and MRI evaluation in goat defects compared to microfracture (Zhang Y. et al., 2018). Intra-articular injection of 25 million human umbilical cord blood-derived MSCs (ubMSCs) and DCECM in OA-induced goats also improved gross articular appearance and radiographic scoring after 6 months (Kim et al., 2022). Additionally, 10 million total human umMSCs and goat chondrocytes in DCECM scaffold encouraged cartilaginous tissue formation with superior glycosaminoglycan content, Young’s modulus, and surrounding tissue integration in caprine chondral defects after 9 months, compared to scaffold alone or nontreatment (Zhang et al., 2020). Importantly, umMSCs persisted in regenerated tissue after 9 months, suggesting sustained chondrogenic differentiation and minimal immunogenicity.

Notably, ruminants rarely develop spontaneous OA and require meniscal removal or disruption—ACL transection alone insufficiently induces significant OA, unlike in other large animals (McCoy 2015). Exercise and weight-bearing protocols are also difficult to implement (Chu et al., 2010), although caprine OA was successfully reported following meniscal manipulation and running regimens (Al Faqeh et al., 2012).

### Equine models

Equine models are uniquely useful yet challenging for cartilage injury investigation. Horses spontaneously develop chondral defects and age/trauma-induced OA secondary to high athletic and lifestyle demands, and thus provide a uniquely-representative model of osteochondral injury for humans (McIlwraith et al., 2012). Surgical OA models can also be generated *via* osteochondral defects, commonly within the carpal joint (McCoy 2015). Equine articular cartilage possesses minimal intrinsic repair capability, with cartilage thickness ranging between 1.5–2 mm and study defect sizes between 6–20 mm (McIlwraith et al., 2012). Horse stifle structure and bone mineral density are also comparable to that of humans (Ahern et al., 2009). Importantly, equine cartilage experiences significantly greater loading forces compared to humans (Murray et al., 2001), which can induce long-term healing failure and is difficult to prevent (McCoy 2015). Horses are also especially costly to maintain due to specialized housing and care.

Allogenic MSCs yield mixed results in equine osteochondral defects. Scaffold-free implantation of 50 million sMSCs into femoral condyle osteochondral defects bolstered gross, MRI, and histological scores of cartilage regeneration (Murata et al., 2022). However, polycaprolactone and HA scaffolds containing bMSCs and cartilage progenitor cells yielded poor neocartilage formation, hypothesized to stem from early implanted cell loss (Mancini et al., 2020). Insufficient MSC numbers and off-target differentiation may also hinder regeneration; implantation of autologous bone marrow concentrate (containing non-expanded bMSCs) and thrombin into full-thickness trochlear defects yielded fibrocartilaginous tissue after 1 year, comparable to microfracture (Chu et al., 2018). Future equine studies may elucidate mechanisms of treatment failure including local inflammation, pathologic scaffold degradation, and MSC dedifferentiation (Mancini et al., 2020).

## MSC sources for articular cartilage regeneration in large animals

MSCs are multipotent, self-renewing progenitor cells isolatable from various tissues and studied for cartilage regeneration *in vitro* (Fulber et al., 2016; Sasaki et al., 2018), in the aforementioned models, and in clinical trials (Matas et al., 2019; Dilogo et al., 2020). Attractively, MSCs can proliferate and differentiate into chondrocytes under endogenous/exogenous signals including matrix, growth factors, proteins, drugs, and mechanical stimuli (Le et al., 2020). MSCs also secrete a secretome of bioactive molecules, i.e., growth factors, prostaglandins, and extracellular vesicles, which modulate the local niche to attenuate inflammation and promote host cell migration, proliferation, differentiation, and matrix deposition (Maumus et al., 2018).

MSCderived exosomes—a subtype of extracellular vesicles—are particularly promising for cell-free cartilage therapeutics. Exosomes are increasingly considered the primary secretory mechanism by which MSCs modulate local healing (Toh et al., 2017), and mediate intercellular communication by exhibiting target-specific paracrine effects on recipient cells. Exosome contents include proteins, nucleic acids, lipids, and other biomolecules encapsulated within phospholipid bilayer and surface ligands (Bao and He 2021). Importantly, exosomes avoid potential shortcomings of direct MSC transplantation, include dedifferentiation, immunogenicity, and batch heterogeneity (Bao and He 2021).

The different MSC tissue sources studied in the aforementioned models (Table 1) affect the proliferation, cartilaginous matrix deposition, chondrogenic differentiation, and overall therapeutic behavior of isolated MSCs (Yaneselli et al., 2018; Gugjoo et al., 2019). bMSCs are among the earliest and most-studied MSC populations in large animal and clinical studies (Lo Monaco et al., 2018). Equine bMSCs exhibit greater chondrogenic potential than aMSCs, while human bMSCs exhibit less calcification potential than sMSCs (Sasaki et al., 2018). However, bone marrow extraction is invasive and complicated by low bMSC density (∼7800 MSCs/ml in humans) (Hernigou et al., 2021). Therefore, aMSCs (particularly from joint-associated adipose) are increasingly popular for cartilage regeneration due to highly-available and easily-accessible tissue, rapid procurement, and chondrogenic potential (Zhang et al., 2019; De Francesco et al., 2021). Ovine aMSCs proliferated faster than bMSCs but expressed lower cartilage-specific gene levels for collagen II, SOX9, and aggrecan *in vitro*, despite both MSC types performing similarly in osteoarthritic ovine knees (Ude et al., 2014). Canine infrapatellar fat aMSCs also exhibited greater proliferation and colony expansion *in vitro* than those from subcutaneous fat (Sasaki et al., 2018). Moreover, equine intra-articular fat aMSCs displayed greater chondrogenic potential than those from non-joint-associated adipose (Gugjoo et al., 2019).

MSCs are exciting for cartilage regeneration due to their significant chondrogenic potential reported by numerous studies. sMSCs are isolatable from synovial membrane or fluid, although equine studies demonstrated superior chondrogenic potential of synovial fluid sMSCs *in vitro* (To et al., 2019). MSCs from subtypes of porcine synovium (fibrous vs. adipose) also varied in growth factor signaling and membrane receptors (Siengdee et al., 2020). Compared with bMSCs and aMSCs, canine sMSCs exhibited superior proliferation, matrix deposition, and rates of stem cell marker CD90 positivity (Sasaki et al., 2018), while porcine sMSCs displayed greater chondrogenic potential (Nakamura et al., 2012). A notable disadvantage is limited synovium and cell quantity—autologous sMSCs are typically expanded prior to reimplantation (To et al., 2019).

Xenogenic human umbilical cord-derived MSCs provide valuable opportunities to study human MSC behavior within large animal osteochondral microenvironments. MSCs are extracted from umbilical cord matrix and blood (Rakic et al., 2018) and demonstrate marked advantages of phenotypic homogeneity, minimal immunogenicity, and tissue availability (Zhang Y. et al., 2018). Simple/noninvasive MSC procurement also circumvents autologous cell extraction procedures and avoids associated donor site morbidity. Promising early findings in multiple large animal models suggest that human umbilical cord MSCs may perform similarly in a clinical setting (Zhang B. Y. et al., 2018; Wu et al., 2019; Kim et al., 2022).

Finally, the choice of autogenic, allogenic, or xenogenic MSCs shapes the conclusions and translatability of large animal studies. Autologous and allogenic mammalian MSCs facilitate proof-of-concept studies, but may yield poor results upon clinical translation due to human and inter-animal/species differences in cell behavior (Siengdee et al., 2020). Subsequently, xenogenic human MSC delivery in large animals helps elucidate human-specific cellular behavior *in vivo* and hint at future translational efficacy, although immunogenicity, interaction with animal host cells, and poor model representation of real-world pathology may still alter outcomes (Lo Monaco et al., 2018; Zayed et al., 2018).

## Tissue engineering strategies for optimizing MSC-based therapies

**FIGURE 1:**
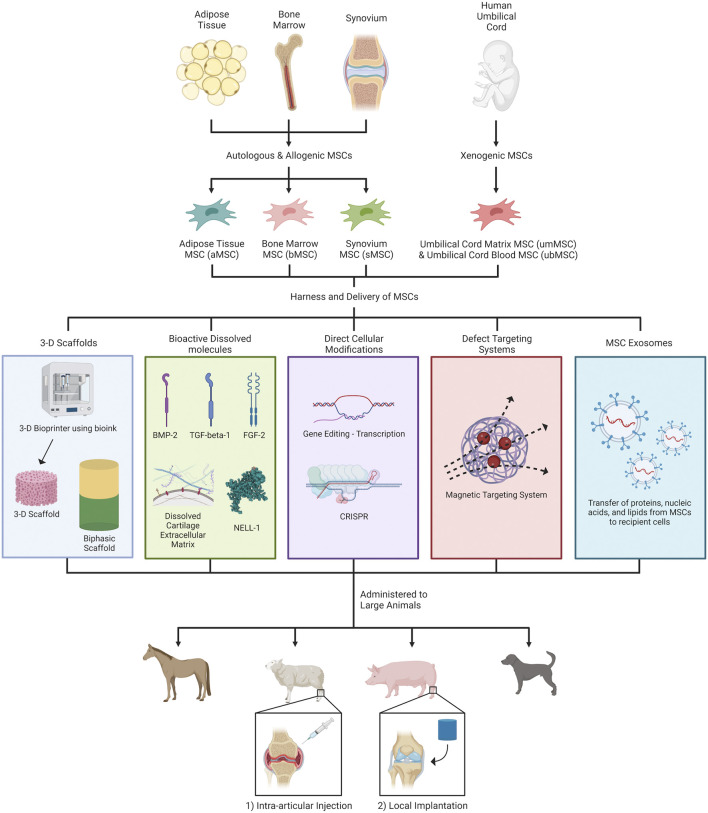
MSC-harnessing strategies for articular cartilage regeneration in large animal models. Different types of MSCs—autologous, allogenic and xenogenic—were first obtained from adipose tissue (aMSC), bone marrow (bMSC), synovium (sMSC), and human umbilical cords (umMSC & ubMSC). Subsequently, MSCs were pretreated with and/or delivered through (1) 3-D scaffolds, (2) bioactive dissolved molecules, (3) direct cellular modifications, (4) defect targeting systems, and (5) cell-free MSC-derived exosomes, for enhancing cartilage regeneration and/or modulating inflammation. Currently, the two major routes of MSC administration in preclinical large animal studies are intra-articular injection and local implantation within chondral/osteochondral defects. Figure created with BioRender.com.

Tissue engineering strategies facilitate MSC delivery and modulate MSC activity, and typically categorize into three-dimensional scaffolds, bioactive dissolved molecules, direct cellular modification, defect targeting systems (Go et al., 2021) and extracellular vesicles (Figure 1 **)**. Several large animal studies report that biphasic osteochondral scaffolds simultaneously promote bone and cartilage regeneration (Zhang et al., 2017; Cunniffe et al., 2019). However, others report mixed results hypothesized to stem from local inflammation and residual byproducts from biphasic construct degradation (Wang et al., 2018; Bothe et al., 2019; Mancini et al., 2020). Alternatively, three-dimensional-printed MSC constructs (Di Bella et al., 2018; Yamasaki et al., 2019) or cell-suspension hydrogels (Levato et al., 2017; Rathan et al., 2019) enable resurfacing of diffusely-osteoarthritic articular surfaces with irregular borders. Bioactive molecules encompass growth factors [bone morphogenic protein-2, transforming growth factor-beta, fibroblast growth factor-2 (Desance et al., 2018), and NEL-like molecule-1 (Li et al., 2016)], matrix molecules including DCECM (Kim et al., 2019; Lu Y. et al., 2021), and ions which recapitulate the native chondrogenic niche and bolster MSC chondrogenic differentiation, proliferation, and matrix expression.

Regarding exosomes, intra-articular injection of 1 mg human MSC exosomes and HA improved cartilage Young’s modulus, stiffness, and MRI/histological scores in porcine femoral condyles (Zhang et al., 2022). Preliminary comparisons, albeit in rodents, also suggest that specific dosages of MSCs and MSC-derived exosomes demonstrate similar cartilage regenerative efficacy (Kim et al., 2020). Although the mechanism is incompletely understood, exosome molecules, i.e., micro ribonucleic acids and enzymes are proposed to upregulate chondrocyte proliferation and chondrogenesis (Toh et al., 2017; Kim et al., 2020). MSC exosomes also reportedly attenuate cartilage injury by inhibiting pathologic inflammation, chondrocyte apoptosis, and macrophage activity (Kim et al., 2020). Nevertheless, exosomes can exhibit nonspecific effects—intra-articular injection of 1 mg human embryonic stem cell exosomes after bone marrow stimulation upregulated bone deposition in porcine chondral defects, with impaired cartilage formation on histology (Hede et al., 2021).

## Conclusion and perspectives

There is a pressing need to develop novel and efficacious therapies for regenerating chondral/osteochondral defects in OA. MSCs are among the most promising substitutes for native chondrocytes, as they can exhibit chondrogenic differentiation and local microenvironment modulation. MSC activity also varies with origin tissue and organism, presenting critical considerations when developing regenerative strategies and interpreting preclinical findings. Comparatively, sMSCs may hold particular promise for future cartilage therapeutic development due to superior chondrogenic potential and clinical accessibility. MSC-derived exosomes may also offer similar efficacy while avoiding direct MSC transplantation.

Given the biomechanical/anatomical differences and potent healing capacity of small animals, preclinical studies must utilize large animal models to adequately develop translatable therapeutics. Notably, most MSC-based studies on cartilage regeneration were conducted in ruminant and porcine models (Table 1; Figure 1) because of anatomical/biomechanical similarity to human tissue, cost-effectiveness, and extensive post-treatment analysis options. Nevertheless, canines and equines also offer unique advantages regarding spontaneous OA modelling and subsequent clinical translatability. Moreover, tremendous advances in chondrogenic MSC delivery systems include architecturally- and mechanically-biomimetic scaffolds (Levato et al., 2017; Rathan et al., 2019) and MSC genetic editing (Sun et al., 2020) (Figure 1).

Despite the limited scope of this mini-review, we determined that there is no clear consensus or standard regarding critical aspects of MSC therapy for large animal cartilage regeneration, particularly regarding effective MSC dosages for implantation or injection in differing species (Table 1) (Li et al., 2018; Kim et al., 2022). Other unstandardized aspects include methodology for MSC chondrogenic induction and/or expansion, surgical protocols for OCG or ACI, and therapeutic benchmarks/controls for cartilage regeneration efficacy in various species. As the field advances, it is paramount to establish comparability between studies and holistically evaluate translatability of MSC-therapeutic findings, especially in large animal models, to maximize clinical relevancy and impact.

## Abbreviations

ACI, autologous chondrocyte implantation; ACL, anterior cruciate ligament; aMSCs, adipose tissue-derived mesenchymal stem cells; bMSCs, bone marrow-derived mesenchymal stem cells; DCECM, decellularized cartilage extracellular matrix; HA, hyaluronic acid; MSCs, mesenchymal stem cells; MRI, magnetic resonance imaging; OA, osteoarthritis; OCG, osteochondral grafting; sMSCs, synovial tissue-derived mesenchymal stem cells; ubMSCs, umbilical cord blood-derived mesenchymal stem cells; umMSCs, umbilical cord matrix-derived mesenchymal stem cells.
